# Adaptation to milking agropastoralism in Chilean goat herders and nutritional benefit of lactase persistence

**DOI:** 10.1111/ahg.12277

**Published:** 2018-09-27

**Authors:** Nicolás Montalva, Kaustubh Adhikari, Anke Liebert, Javier Mendoza‐Revilla, Sergio V. Flores, Ruth Mace, Dallas M. Swallow

**Affiliations:** ^1^ Research Department of Genetics, Evolution and Environment University College London Darwin Building, Gower Street London WC1E 6BT United Kingdom; ^2^ Department of Anthropology, Human Evolutionary Ecology Group University College London 14 Taviton St London WC1H 0BW United Kingdom; ^3^ Departamento de Antropología, Facultad de Ciencias Sociales y Jurídicas Universidad de Tarapacá 384 Calle Cardenal Caro Arica Chile; ^4^ Department of Cell & Developmental Biology University College London Anatomy Building, Gower Street London WC1E 6BT United Kingdom; ^5^ Laboratorios de Investigación y Desarrollo, Facultad de Ciencias y Filosofía Universidad Peruana Cayetano Heredia 430 Honorario Delgado Lima 31 Perú; ^6^ Departamento de Antropología, Facultad de Ciencias Sociales Universidad de Chile 1045 Av. Capitan Ignacio Carrera Pinto Nunoa 7800284 Chile

**Keywords:** Adaptation, body mass index, lactase persistence, Latin America, natural selection, pastoralism

## Abstract

The genetic trait of lactase persistence (LP) evolved as an adaptation to milking pastoralism in the Old World and is a well‐known example of positive natural selection in humans. However, the specific mechanisms conferring this selective advantage are unknown. To understand the relationship between milk drinking, LP, growth, reproduction, and survival, communities of the Coquimbo Region in Chile, with recent adoption of milking agropastoralism, were used as a model population.

DNA samples and data on stature, reproduction, and diet were collected from 451 participants. Lactose tolerance tests were done on 41 of them. The European −13,910*T (rs4988235) was the only LP causative variant found, showing strong association (99.6%) with LP phenotype.

Models of associations of inferred LP status and milk consumption, with fertility, mortality, height, and weight were adjusted with measures of ancestry and relatedness to control for population structure. Although we found no statistically significant effect of LP on fertility, a significant effect (*P* = 0.002) was observed of LP on body mass index (BMI) in males and of BMI on fertility (*P* = 0.003). These results fail to support a causal relationship between LP and fertility yet suggest the idea of a nutritional advantage of LP. Furthermore, the proportion of European ancestry around the genetic region of −13,910*T is significantly higher (*P* = 0.008) than the proportion of European ancestry genome‐wide, providing evidence of recent positive selection since European–Amerindian admixture. This signature was absent in nonpastoralist Latin American populations, supporting the hypothesis of specific adaptation to milking agropastoralism in the Coquimbo communities.

## 1 INTRODUCTION

Lactase, the enzyme responsible for the digestion of lactose in milk, is downregulated after weaning in most mammals, including most humans. Lactase persistence (LP) is a genetic trait that results in continued high levels of lactase activity in some human adults and allows for digestion of milk as part of the adult diet. LP (worldwide frequency ∼35%) is very frequent in some populations, including most of Northern Europe and many East African and Middle Eastern groups, where it is strongly correlated with pastoralist subsistence strategies and milk drinking (Holden & Mace, 1997; Ingram, Mulcare, Itan, Thomas, & Swallow, 2009a; Jones et al., 2015). Several different alleles have been identified as causative of LP in different populations (i.e. there is evidence of convergent evolution) (Enattah et al., 2002; Imtiaz et al., 2007; Ingram et al., 2007, 2009b; Jones et al., 2013; Tishkoff et al., 2007). These variants are located on extended undisrupted haplotypes (Bersaglieri et al., 2004; Liebert et al., 2017; Poulter et al., 2003; Ranciaro et al., 2014; Tishkoff et al., 2007), reflective of their recent origin. The European LP allele (−13010*T, rs4988235) is thought to have increased to the current frequencies during the past 5000 to 10,000 years since animals were first milked (Evershed, Payne, & Sherratt, 2008; Itan, Powell, Beaumont, Burger, & Thomas, 2009) and has rarely been found before 5000 B.P. (see Liebert et al., 2017, Supplementary Figure 3 for map and references). These data support the hypothesis of strong positive natural selection favoring LP in groups that developed milking practices soon after animal domestication. According to most authors, very high selection coefficients (>0.05, which means a 5% increase in progeny for the carriers of that allele) would have been needed, based on current frequencies of LP and the time since animal domestication or the age of the mutations (Aoki, 1991; Bersaglieri et al., 2004; Gerbault, Moret, Currat, & Sanchez‐Mazas, 2009; Itan et al. 2009; Peter, Huerta‐Sanchez, & Nielsen, 2012; Tishkoff et al., 2007). More recently this has been supported by ancient DNA studies, as discussed by Mathieson et al. (2015).

It appears that milk consumption must have been very influential in terms of survival, fertility, or both, but how exactly this operated is much less clear. It is likely that there were different selective pressures in different geographic regions, but the nutritional benefits of milk were most likely the driving force in Europe. A number of studies have reported that people with LP are of greater height or weight than people without LP, suggesting that their consumption of milk does confer a clear nutritional advantage (Almon et al. 2010, Almon, Álvarez‐León, & Serra‐Majem, 2012; Corella et al., 2011; Kettunen et al., 2010; Lamri et al., 2013).

To better understand the relationship between milk drinking, LP, growth, reproduction, and survival, milk‐dependent goat herders from the agricultural communities of the Chilean region of Coquimbo in South America (additional data in Supplementary Material section 1) were used as a model population because of their agropastoralist livelihood and the recent introduction of both animal milking and LP some 400 years ago. This study aimed to assess whether there was any evidence for differential weight, height, fertility, and survival in people with LP while also interrogating genetic data for evidence of selection. If selection of the magnitude reported has been ongoing in recent generations, it might be detectable by examining the proportions of European ancestry in the lactase gene region in relation to genome wide ancestry, as has been done for other loci in admixed Latin American samples (Rishishwar et al., 2015; Zhou, Zhao, & Guan, 2016) and for genes involved in adaptation to hypoxia in admixed Tibetan populations (Jeong et al., 2014).

## 2 MATERIALS AND METHODS

### 2.1 Subjects

The study volunteers were recruited from the agricultural communities of the Chilean region of Coquimbo in South America (see Alexander, 2008, Gallardo, 2002, Vergara, Toro, Bonilla, & Meneses, 2005 and Supplementary Material sections 1 and 2 for further details.)

### 2.2 Data collection

A total of 451 adult volunteers were recruited from nine villages and hamlets in the Coquimbo region. Data collection methods, questionnaire details, and a demographic profile based on this sample can be found in Supplementary Material section 2. Data on number of children were those self‐declared by the parents at interview, thus with some inherent inaccuracy. The height and weight of the participants were measured using a portable scale and stadiometer.

Lactose digestion phenotype was determined, as a surrogate for LP phenotype, by lactose tolerance testing using the breath hydrogen method as described by Ingram et al. (2007) and in Supplementary Material section 2. Lactose digesters were those who showed no substantial rise in breath hydrogen after 3 hours; those with a maintained rise above 20 ppm were classified as nondigesters. Participants showing varying levels of breath hydrogen that did not stabilize above 20 ppm were classified as indeterminate, and those who failed to produce breath hydrogen throughout the test were classified as hydrogen nonproducers (i.e. do not have appropriate hydrogen‐producing colonic bacteria).

### 2.3 DNA methods

Samples of buccal cells were used as a source of DNA and used for sequencing 706 bp of the *LCT* enhancer region (*MCM6*, intron 13). In addition, each individual was also typed for a set of 15 autosomal short tandem repeats (STRs), 30 single nucleotide polymorphisms (SNPs) used as Ancestry Informative Markers (AIMs), and 27 SNPs in chromosome 2 covering the 1.77 Mb region surrounding −13,910C > T, to be used for haplotype inference and estimations of whole‐genome and local ancestry (see Supplementary section 3). A previous study conducted in Latin Americans (Ruiz‐Linares et al., 2014) has shown a 70% correlation of ancestries deduced from these 30 AIMs with those estimated from ∼50,000 genome‐wide SNPs (after LD pruning). The 27 SNPs in chromosome 2 included two polymorphisms (rs3754689 and rs2278544) useful for identification of the core haplotypes described by Hollox et al. (2001). Details of the genotyped markers can be found in Supplementary Material section 3.

### 2.4 Analysis methods

PHASE 2.1.1 (Stephens & Donnelly, 2003; Stephens, Smith, & Donnelly, 2001) was used to infer 1.77 Mb haplotypes using the 27 markers surrounding −13,910C > T plus the genotypes for −13,910C > T (rs4988235) obtained from sequencing to make a total of 28. A dataset of 190 individuals from different Old World populations genotyped for the same variants (Liebert et al., 2017) was included in this analysis to assess the ancestral origin of the haplotypes carrying the −13,910*T allele by comparison.

The AIMs data were merged with reference panels containing European CEU (Utah residents from the collection of the Centre d'Etude du Polymorphism Humain) and African YRI (Yoruba in Ibadan, Nigeria) from The 1000 Genomes Project Consortium (2012) and Amerindian samples (Ruiz‐Linares et al., 2014). These were used for supervised (*k *= 3 components) runs of the clustering program Admixture (Alexander, Novembre, & Lange, 2009) for measuring the proportions of continental ancestry genome wide. Because of the very low (<0.05) estimated proportion of African ancestry, Admixture was also used without Africans as one of the parental populations, both supervised and unsupervised, *k *= 2 for analysis of the AIMs. For the unsupervised (*k *= 2) run, the reference European and Amerindian samples had averages of 96% and 99% for their respective ancestry components.

To conduct Local Ancestry analysis (i.e. compare ancestry proportions surrounding a specific genomic region with genome‐wide ancestry proportions (*sensu* Falush, Stephens, & Pritchard, 2003, Padhukasahasram, 2014) at this genomic region, the chromosome 2 data were merged with the reference panel containing the same European, African, and Amerindian reference samples as discussed. After merging, a set of 16 SNPs, of the original 28 SNPs, LD pruned with PLINK (r^2 ^< 0.5) (recommended settings from Purcell et al., 2007), was retained for further analysis (see Supplementary Material section 3). Supervised ancestry estimates using Admixture for this genomic region were compared with the genome‐wide ancestry assessed using the AIMS. The difference, or “Δ–ancestry,” in European continental ancestry for this genomic region from the genome‐wide estimate (Tang et al., 2007) was calculated for each individual. A nonparametric Wilcoxon signed‐rank test was applied to test for a significant difference between the pairwise (i.e. across each individual) distributions of local vs genome‐wide European ancestry. A one‐sided hypothesis was tested of whether the European local ancestry distribution is greater in value than the genome‐wide ancestry distribution. Power calculations were performed based on simulations. (Refer to supplementary Material section 5 for more detailed information.)

To test for inbreeding, an average value of *F_is_* (*F*) was calculated for each individual based on 1000 values sampled from the likelihood distribution of homozygosity of the 15 STRs genotyped. To compare these values, the same procedures were used with a dataset of 153 African individuals (62 Jaali and 91 Somali), data already available in the lab, and previously reported as unrelated at the grandparental level and genotyped at the same loci (Ingram et al., 2007, 2009b). A further indicator of relatedness, namely the proportion of shared STR alleles (PSA) (Cardoso, Lau, Eiras Dias, Fevereiro, & Maniatis, 2012; Chakraborty & Jin, 1993; Zhao et al., 2007), was also determined.

Multiple regression models used to explore the effect of LP on stature and fertility were adjusted for nongenetic and genetic variables as described in the Results section. For the STRs, membership of clusters was taken from an unsupervised run of STRUCTURE (Falush et al., 2003, Falush, Stephens, & Pritchard, 2007; Hubisz, Falush, Stephens, & Pritchard, 2009; Pritchard, Stephens, & Donnelly, 2000) and determining the best value for *k* using the method described by Evanno, Regnaut, and Goudet (2005). For the mixed‐effect models, a PSA matrix was also incorporated.

Because each analysis uses different numbers of genetic markers and different demographic data and can be done over a different sample size, a summary of samples and data used for each analysis is presented in Supplementary Material section 6.

## 3 RESULTS

### 3.1 Genotype–phenotype association

Altogether data were collected from 451 participants. Of these, 41 were phenotyped for lactose digestion; 19 participants were classified as lactose digesters and 17 as nondigesters (Table 1). Lactose digestion status could not be determined for 5 participants (4 indeterminate and 1 hydrogen nonproducer).

**Table 1:** Association of lactose digestion phenotypes obtained from lactose tolerance tests (LTTs) with −13,910 C > T genotype. According to a full dominance model, homozygotes for the −13,910*C allele (CC) are predicted non‐digesters, while homozygotes for the −13,910*T allele (TT) and heterozygotes (CT), are digesters

LTT phenotype	CC	CT	TT	Total
Nondigesters	17	0	0	17
Digesters	1	16	2	19
Indeterminate	3	0	1	4
H_2_ nonproducer	1	0	0	1
**Total**	**22**	**16**	**3**	**41**

Complete sequencing of the enhancer region showed that 18 of the 19 digesters carried the European variant causative of LP, −13,910*T, but none of the nondigesters did. No other LP variants or unreported variants were found from the examination of the sequences. Phenotyped digester status was highly associated with predicted LP status according to −13,910C > T genotype (CT + TT considered as digesters, Fisher's exact test, *P *< 0.001).

### 3.2 Allele frequencies and haplotypic background of −13,910*T alleles

Samples from a total of 437 of the 451 collected were successfully sequenced, and it was possible to determine the presence of the European −13,910*T allele, at a frequency of 0.22, and absence of any other polymorphic sites known or likely to be causal of LP. This is a slightly lower frequency than that of 0.27 found in the subset of samples in Table 1. Genotype frequencies for this locus, rs4988235 (reported in Fernández et al., 2016), were in agreement with expectations from Hardy‐Weinberg equilibrium (χ^2^ = 1.15; *P* >0.05).

Haplotype analysis of the 1.77 Mb chromosome 2 region identified 624 distinct haplotypes (See Supplementary Material, figure S4.1) and showed that 90% of −13,910*T alleles are associated with a European‐like extended **A** core haplotypic background of greater than 900 Kb in length, confirming −13,910*T as a European introduction. There was no evidence of population differentiation with respect to rs4988235 across the nine communities (Fisher's exact test, genotypic *P* = 0.4215; allelic *P* = 0.366). Supplementary Material figure S4.2 shows examples of data separated by village and the lack of significant substructure.

### 3.3 Relatedness

To control for relatedness, a possible problem resulting from the small size of these populations, 15 autosomal STRs were used. Cases with incomplete genotyping were removed, resulting in a final sample of 351 individuals used for the analyses. All 15 markers showed high variability, and there was no significant difference between expected and observed heterozygosity over all loci (*t*‐test, *P* = 0.8264). Across loci, the observed variance in heterozygosity was similar to the expected variance (Bartlett test of homogeneity of variances K^2^ = 0.1742; *P* = 0.6764) (Barlett, 1954; Evans, Bartlett, Sweijd, Cook, & Elliott, 2004).

Average values of *F_is_* calculated for each individual (see Methods) resulted in values of *F_is_* ranging from 0.071 to 0.611, with a mean of 0.149 and a standard deviation (SD) of 0.07. In the African dataset of individuals reported as unrelated at the grandparental level, *F_is_* ranges from 0.072 to 0.471, with a mean of 0.151 and an SD of 0.072. Both datasets show similar positively skewed distributions, with a longer tail in the Chilean dataset caused by outliers with high *F_is_*, but differences in these distributions are not significant (two samples Kolmogorov–Smirnov test; *P* = 0.913; see Keller & Arcese, 1998 for an example of this analysis), suggesting that inbreeding in the Chilean samples is not significantly higher than inbreeding in the reportedly unrelated African individuals.

### 3.4 Ancestry

Genotypes of 30 AIMs were obtained for all 437 individuals, but those with too many missing genotypes (>20% failure rate) were removed, resulting in a final sample size of 408 individuals used for the analyses. None of the 30 loci deviated significantly from Hardy‐Weinberg equilibrium (*P* > 0.05). These markers were used to estimate proportions of European, African, and Amerindian ancestry for each individual, according to their similarity to a reference dataset of 876 individuals from three parental populations: 299 Europeans, 169 Africans, and 408 Amerindians (obtained by Ruiz‐Linares et al., 2014).

**Figure 1:**
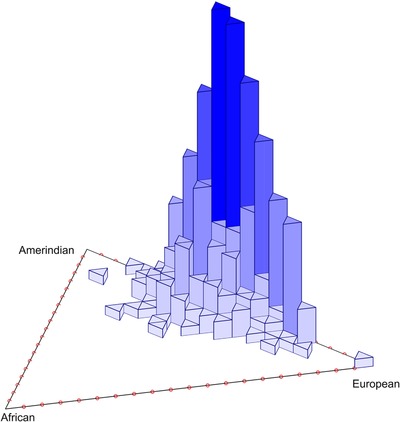
Histogram of estimated ancestry proportion from three parental populations. European, African, and Amerindian using Admixture 1.23. The peak number of individuals (vertical axis) is concentrated between European and Amerindian ancestry, with little contribution from Africa [Color figure can be viewed at wileyonlinelibrary.com]

The distribution of estimated ancestry proportions can be examined visually in a trivariate histogram (Ruiz‐Linares et al., 2014, Supplementary Text S1), showing a wide range of Amerindian–European admixture, with marginal African contribution (Figure 1). The proportion of Amerindian ancestry gives a mean of 0.47 (s = 0.12; min–max = 0–0.8), and the proportion of European ancestry has a mean of 0.48 (s = 0.13; min–max = 0.09–1). In contrast, the proportion of African ancestry is only 0.05 on average (s = 0.06; min–max = 0–0.29). There was no significant difference in the mean proportion of European ancestry across the communities (one‐way analysis of variance [ANOVA]; *P* = 0.51; see Supplementary Material section 4 for more information).

### 3.5 Assessment of population stratification of −13,910*T carriers by relatedness and ancestry

**Figure 2:**
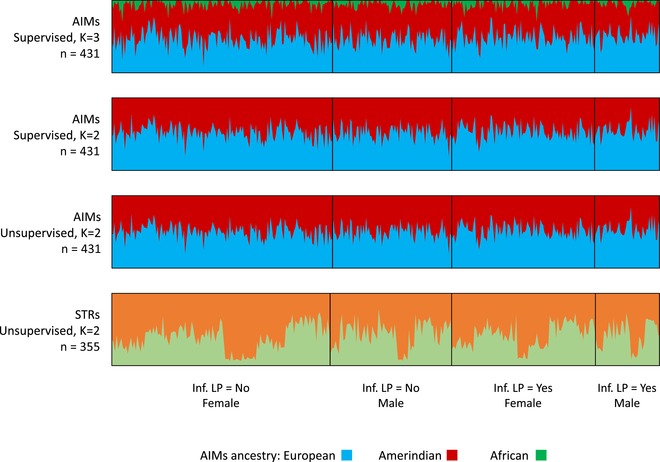
Results of cluster analysis according to inferred lactase persistence (LP) status and sex. Inf. LP = Inferred lactase persistent. No statistically significant difference was found between LP and nonpersistence (LNP) status in the Ancestry Informative Markers (AIMs) clustering. The proportion of European ancestry obtained by supervised Admixture analysis for *k *= 3 gives LP = 0.5, LNP = 0.47, *t‐*test *P* = 0.058. The average proportion of European Ancestry (blue) for *k* = 2 unsupervised is LP = 0.53; LNP = 0.51; *t‐*test *P* = 0.135. Note that by clustering at *k *= 2 for ancestry, the small African component seen in Figure 1 is not detected. For the single tandem repeat (STR) markers (lower panel) the colors simply reflect the two clusters for *k *= 2 obtained by STRUCTURE, and although there are village level differences in the clustering of the STRs (Figure S4.2), there are not between LP and LNP [Color figure can be viewed at wileyonlinelibrary.com]

Relatedness and ancestry were both examined in relation to predicted LP status to evaluate the importance of the confounding effect of these sources of population structure. This was done using an unsupervised analysis, both by clustering the STR markers and the AIMs (Figure 2) and using inferred LP status as though LP and LNP were two different populations, taking −13,910*T as causal of LP and a model of full dominance of the causal allele. Neither test indicated spurious stratification of the two phenotypic groups. Additionally, neither estimated values of *F_is_* nor the proportion of European ancestry as measured using the AIMs with Admixture are significantly different between inferred lactase nonpersistent and inferred lactase persistent groups (*F_is_* LNP = 0.14; *F_is_* LP = 0.15, *t‐*test *P* = 0.25; European ancestry LP = 0.5, NLP = 0.48, *t‐*test *P* = 0.062).

### 3.6 Local ancestry and lactase persistence

Local ancestry assignment at the *LCT* region was conducted in two different ways after removing an individual of recent European origin. Because the derived allele (−13,910*T) for the causal SNP, rs4988235, is absent in both African and Amerindian parental populations, a supervised run of Admixture, grouping African and Amerindian reference samples into a “non‐European” reference group, was performed in which the two reference groups were labeled European and non‐European. A second supervised run of Admixture excluding African reference samples, as well as Chilean goat herders with >1% African ancestry was also performed with only European and Amerindian reference groups. European local ancestry estimates for both admixture runs were consistent. Similar admixture runs were done (after LD pruning) for SNPs in the 1.77 Mb region housing *LCT* on chromosome 2.

“Δ–ancestry,” the difference in European continental ancestry estimates for the *LCT* region and the genome‐wide estimate, was 3% for both the first (n = 433) and second (n = 163) runs. This is consistent with the idea that if there is recent positive selection for the European allele at rs4988235 in the Chilean goat herders since admixture, the difference between local and genome‐wide ancestry should be positive for most individuals. The differences were significant in both cases, with *P* values of 0.0082 and 0.0095, respectively, for the two runs (one‐sided Wilcoxon signed‐rank test).

To contrast this analysis with urban nonpastoralist Latin American populations sampled in the 1000 Genomes Phase 3 (The 1000 Genomes Project Consortium 2012), the analysis was repeated with the MXL (Mexicans from Los Angeles, USA; n = 55) and PEL (Peruvians in Lima, Peru; n = 76) population groups, the two other groups with low levels of African ancestry according to 1000 Genomes Project. The same set of 16 SNPs and the same AIMs were extracted from the 1000 Genomes database, and a supervised run of Admixture with the two reference groups, European and Amerindian, was performed on both populations. The Wilcoxon test for enrichment of European local ancestry was not significant for either groups, with *P* values of 0.5632 for MXL and 0.9138 for PEL. This analysis was further validated for all three mainland Latin American groups from 1000 Genomes: MXL, PEL, and CLM (Colombians from Medellin, Colombia; n = 93) using the available whole‐genome high‐density genotype data. (See Supplementary section 6 for further details and power calculations.)

### 3.7 Lactase persistence and milk consumption

Estimated consumption of fresh milk using number of glasses of fresh milk per day gives a relatively low average (0.57 glasses per day) but with a high SD (±0.74 glasses). This variation does not seem to be related to LP and showed no significant association with genotypes (mean CC = 0.55, mean CT = 0.52, mean TT = 0.72, ANOVA *P* = 0.47), with inferred LP status (mean digester = 0.55, SD = 0.7; mean nondigester = 0.55, SD = 0.7; *t‐*test *P *= 0.94), or with lactose digester status based on lactose tolerance tests (mean digester = 0.54, mean nondigester = 0.54, *t‐*test *P *= 0.99).

Similar results were found in the analysis of consumption of milk products, which contain variable amounts of lactose, with no association between avoidance of milk products and genotype (χ^2^ *P* = 0.4), inferred digester status (Fisher's exact test, *P* = 0.92), or lactose digestion (Fisher's exact test, *P* = 1).

### 3.8 Lactase persistence and body mass index

Multiple regression analysis was used to examine the association between inferred LP and body size using BMI and height as response variables. Explanatory variables included in the model were lactose digestion status (based on −13,910C > T genotype), age, glasses of milk consumed per day, proportion of measured European ancestry (as assessed from *k = 2*), wealth, inbreeding coefficient (*F*_is_), and proportion of assignment to one cluster of a *k = 2* run of STRUCTURE based on STRs. Additionally, the age at first birth variable was included for females (as an indicator of growth cessation). A mixed‐effects model (Gałecki & Burzykowski, 2013; Henderson, 1984), additionally using the PSA matrix to adjust for kinship, as found to be a powerful by Cardoso et al. (2012), was also run. The single apparently 100% European outlier was removed for these analyses. Table 2 shows the effects on BMI and height in these multiple regression analyses, and Supplementary Figure 4.4 shows the effect on BMI at the village level.

**Table 2:** Effects of predicted lactase persistence (LP) and lactose digestion status on height and body mass index (BMI). Models also adjusted for age, milk consumption, proportion of European ancestry, wealth, inbreeding coefficient (*F*), and relatedness or consanguinity. Additionally, the age at first birth variable was included for females

Height:									
				Inferred LP†		Proportion European ancestry		Age	
Group	n	log‐likelihood	R^2^	β	*P*‐value	β	*P*‐value	β	*P*‐value
Males (Lm‡)	110	−371.65	0.08	1.102	0.47	5.083	0.43	−0.070	0.081
Males (Mx§)	110	−371.65	–	1.102	0.45	5.084	0.41	−0.070	0.067
Females (Lm)	188	−581.32	0.18	−0.324	0.70	−0.006	1.00	−0.137	**<0.001**
Females (Mx)	188	−581.33	–	−0.324	0.69	−0.006	1.00	−0.137	**<0.001**
Both (Lm)	330	−1074.47	0.50	0.400	0.58	2.157	0.46	−0.120	**<0.001**
Both (Mx)	330	−371.65	0.08	1.102	0.47	5.083	0.43	−0.070	0.081

BMI:									
				Inferred LP		Proportion European ancestry		Age	
Group	n	log‐likelihood	R^2^	β	*P*‐value	β	*P*‐value	β	*P*‐value
Males (Lm)	110	−285.05	0.09	1.998	**0.0047**	−0.482	0.87	0.019	0.31
Males (Mx)	110	−285.05	–	1.998	**0.0027**	−0.482	0.86	0.019	0.29
Females (Lm)	187	−550.07	0.06	0.446	0.54	−6.526	0.021	0.012	0.61
Females (Mx)	187	−550.08	–	0.446	0.53	−6.525	0.017	0.012	0.60
Both (Lm)	329	−957.32	0.51	0.998	0.057	−4.728	0.023	0.013	0.36
Both (Mx)	327	−952.24	–	1.010	0.049	−4.772	0.020	0.013	0.37

^†^Inferred LP from rs4988235 (−13010C > T genotype).

^‡^Lm : linear model. Variance inflation factor (VIF) was < 1.1 in all cases.

^§^Mx : mixed model. Variance inflation factor (VIF) was < 1.1 in all cases.

**Figure 3:**
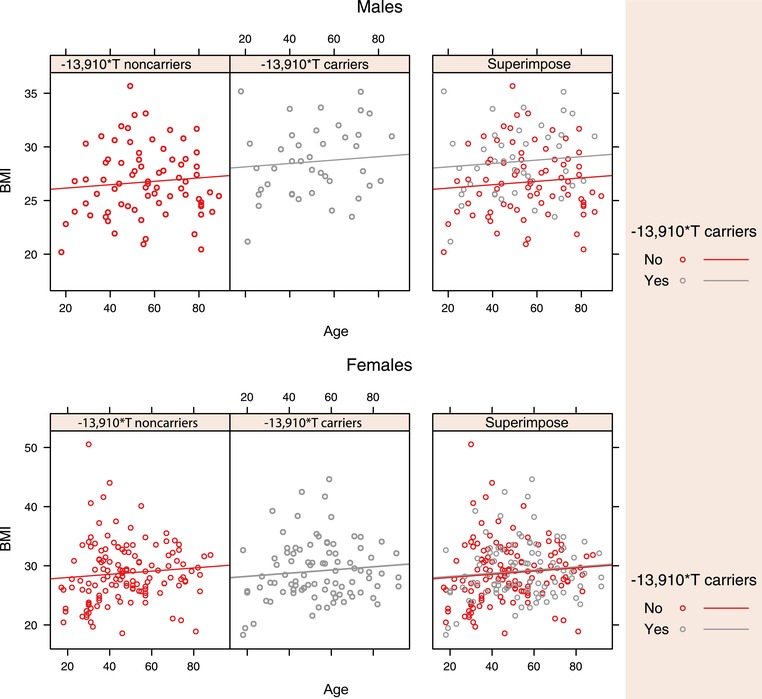
Comparison of increment of body mass index (BMI) with age between −13,910*T carriers in males and females. There is a significant difference in BMI between inferred lactase persistent (LP) (T allele carriers: TT and CT) and inferred non–lactase persistent males (LNP) C allele homozygotes (CC). This trend remains significant adjusting for age, milk consumption, ancestry, wealth, inbreeding, and relatedness. However, there is no significant difference in BMI between inferred LP and non‐LP females [Color figure can be viewed at wileyonlinelibrary.com]

Inferred LP status showed a significant effect on BMI in males, increasing it by 1.998 kg/m^2^ ± 0.691 Standard Error (SE) (*t* = 2.892, *df* = 102, *P* = 0.0047) but no significant effect on BMI in females nor on height in any of the models. BMI in females is significantly affected by the proportion of European ancestry, which for each percentile decreases by 0.065 kg/m^2^ ± 0.028 SE. Also, age at first birth affects BMI; each extra year decreases BMI by 0.139 kg/m^2^ ± 0.068 SE The only variable with significant effect on height was age, which decreases height in both sexes. The BMI versus age plot in LP and LNP females and males is shown in Figure 3. It is somewhat surprising that the proportion of ancestry was not correlated with height in this data set, unlike in other mixed Latin American data sets (e.g. Ruiz‐Linares et al., 2014).

The mixed‐effects regression model identified the same fixed effects as significant, but the *P* value of each variable was slightly lower than in the model without inclusion of PSA matrix as random effects. Together these results showed a greater BMI in inferred LP (T allele carriers) compared with non‐LP males. This effect is statistically significant even after controlling for age, milk consumption, ancestry, wealth, inbreeding, and relatedness (Table 2). Inferred LP does not have a significant effect on size in females in either model.

### 3.9 Lactase persistence and number of children

Differences between inferred LP and inferred non‐LP individuals in number of children ever born (CEB) and number of surviving children were examined to identify whether there was any direct evidence of ongoing differences in fitness between the two groups. Because of the general trend toward lower mortality rate because of demographic transition, deaths of children were rare events in this sample and occurred almost exclusively for participants born before 1970. Only 14% of the female participants reported to have experienced the death of a child.

The low child mortality rate makes it difficult to compare the number of child deaths between inferred LP and non‐LP groups. We therefore compared the ratio of CEB and of surviving children in both groups, and this showed no significant difference (*t*‐test: *df* = 37.7, *P* = 0.12). (Cox proportional hazards: n = 1261 children, number of casualties = 85, *P* = 0.53.)

Additional analyses were done based only on number of CEB, and in this case, inferred digesters have 2.92 children on average, and inferred nondigesters have an average of 2.94, a difference that is not significant (*t*‐test: *df* = 341.22, *P* = 0.86). However, for this sample size to detect with a power of 0.9, any difference in average number of children would have to be greater than 0.4.

Zero‐inflated regression, a generalized model of count data with a high frequency of zeros, was used to model the effect of LP on the number of CEB and number of surviving children, with explanatory variables LP status, as well as sex, age, wealth, glasses of milk consumed per day, and BMI (Table 3). There is no evidence of an effect of inferred LP status on number of children and therefore no direct evidence of improved fitness in LP individuals from this dataset. But interestingly, the effect of BMI on total number of CEB is statistically significant (*P* = 0.002) for the whole group, including both sexes, after controlling for the other variables.

**Table 3:** Zero‐inflated regression model tested for number of children ever born and number of surviving children

	Children ever born			Surviving children		
Covariate	β	SE	*P*‐value	β	SE	*P*‐value
Digester (yes)	−0.053	0.063	0.395	−0.050	0.065	0.447
Sex (male)	−0.109	0.070	0.117	−0.075	0.074	0.312
Age	0.025	0.002	**<0.001**	0.023	0.002	**<0.001**
Body mass index	0.022	0.007	**0.002**	0.022	0.007	**0.002**
European ancestry	−0.009	0.242	0.971	−0.154	0.255	0.547
Glasses of milk per day	−0.013	0.041	0.746	−0.016	0.043	0.714
Wealth	−0.212	0.213	0.321	−0.225	0.222	0.312

## 4 DISCUSSION

The evidence for strong positive natural selection favoring LP in the past in some populations is overwhelming, but the specific mechanisms are unknown. There has been much discussion about the advantages of calcium absorption at high latitudes and water content in arid environments, as well as others not related to milk consumption (Gerbault et al., 2009; Sverrisdóttir et al., 2014). This study aims to obtain evidence of ongoing selection and better understand possible mechanisms using the agropastoralist communities of goat herders from Chile as a model population.

We have confirmed the introduction of European haplotypes carrying the variant −13,910*T (rs4988235) into this group and shown that about 40% of the population are lactase persistent. We found that individuals homozygous for −13,910*C, who are inferred to be lactase nonpersistent, did not consume significantly less lactose than lactase persistent individuals, suggesting no avoidance of milk caused by symptoms of milk intolerance. However, a significant effect of LP on BMI was found in this population, indicating that males carrying −13,910*T might obtain more nutritional benefit from the same amount of milk. It should be noted that this benefit could come from additional components of the milk, as well as lactose, because noncarriers are more likely to develop diarrhoea, potentially compromising uptake of other nutrients because of damage to their small‐intestinal epithelium.

The findings reported here agree with previous work showing an association between LP and obesity in European populations (Almon et al. 2010; Corella et al., 2011; Kettunen et al., 2010; Lamri et al., 2013; Malek, Klimentidis, Kell, & Fernández, 2013) and admixed Latin American populations (Hartwig, Horta, Smith, de Mola, & Victora, 2016). However, in the present study, an increment in BMI was only observed in males, which might possibly be caused by sex biases in feeding practices for children in these communities, a practice reported in several rural communities worldwide (Chen, Huq, & D'Souza, 1981; Khera, Jain, Lodha, & Ramakrishnan, 2014). An increment in BMI could be considered on its own to be an evolutionary advantage whenever there are episodes of food shortage or famine, protecting against life‐threatening weight loss, but could result in obesity in populations where food is abundant. It should be noted that LP is not overrepresented among obese individuals in our study (defined as BMI > 30 kg/m^2^, Fisher's exact test, *P* = 0.2; see Figure 3).

In this study, we have also found a significant effect of BMI on number of children, as reported in other studies (Power & Schulkin, 2008; Speakman, 2006; Weng, Bastian, Taylor, Moser, & Ostbye, 2004), but the model does not show a statistically significant effect of LP on fitness mediated through BMI. This may simply be due to a lack of power because the numbers of child deaths in our sample was very low.

The significant enrichment for European ancestry in the Chilean goat herders is, however, suggestive of positive selection for the European allele −13,910*T at rs4988235 since admixture. Although the SNP set was small and a haplotype‐based method for determining local ancestry might have been preferable, the effect we observed here was not found in urban nonpastoralist Latin Americans either as tested using the same SNPs. No published genome‐wide studies have reported a signal of recent selection of the *LCT* region in other admixed Latin American populations, and our own attempts to find that using public genome‐wide data for other Latin American populations also failed to show this (see Supplementary section 5). Although stochastic effects causing this pattern in the goat herders cannot be excluded, these results support the notion that the effect we observe is indicative of recent selection (i.e. since admixture) in this population caused by adoption of milking and milk dependence, and it is consistent with the idea that LP offers a way to gain weight. We propose that this conferred a selective advantage in reproductive fitness and survival, which would have been more evident before the recent demographic transition and in the context of famine or low food availability.

## Supporting information

Table S1. Population of sampled sites, total population, and number of participants in the study (n).
Figure S1.
Table S2.1. Demographic profile of 451 sample donors from nine villages.
Table S2.2. Sample information by sex.
Figure S2.1. Percentage of participants by sex in each village.
Figure S2.2. Age of participants by sex.
Figure S2.3. Proportion of European ancestry of participants by sex.
Figure S2.4. Height of participants by sex.
Figure S2.5. Weight of participants by sex.
Figure S2.6. BMI of participants by sex.
Figure S2.7. Milk consumption of participants by sex.
Figure S2.8. Children ever born of participants by sex.
Table S3.1. Twenty‐seven SNPs surrounding *LCT* enhancer region on chromosome 2, genotyped for haplotype inference.
Table S3.2. Thirty Ancestry Informative Markers (AIMs), genotyped for ancestry estimations.
Table S3.3. Fifteen highly variable autosomal single tandem repeats, genotyped for estimations of relatedness.
Figure S4.1. Diversity and frequency of the 624 distinct 1.77‐Mb haplotypes deduced by PHASE (874 chromosomes).
Figure S4.2. Analysis of STRUCTURE at village level for both Ancestry Informative Markers (above: supervised analysis at *k* = 3: green: African; red: Amerindian; blue: European) and single tandem repeat markers (below).
Figure S4.3. Counts of rs4988235 genotypes per village.
Figure S4.4. Boxplot of body mass index per village by sex.
