# Supplementary material for: Adaptation to milking agropastoralism in Chilean goat herders and nutritional benefit of lactase persistence
Source: Ann Hum Genet. 2018 Sep 27;83(1):11–22. doi: 10.1111/ahg.12277 (PMC6393766; doi:10.1111/ahg.12277)
Supplement: Supplementary file 1 — Table S1. Population of sampled sites, total population, and number of participants in the study (n). Figure S1. Table S2.1. Demographic profile of 451 sample donors from nine villages. Table S2.2. Sample information by sex. Figure S2.1. Percentage of participants by sex in each village. Figure S2.2. Age of participants by sex. Figure S2.3. Proportion of European ancestry of participants by sex. Figure S2.4. Height of participants by sex. Figure S2.5. Weight of participants by sex. Figure S2.6. BMI of participants by sex. Figure S2.7. Milk consumption of participants by sex. Figure S2.8. Children ever born of participants by sex. Table S3.1. Twenty‐seven SNPs surrounding LCT enhancer region on chromosome 2, genotyped for haplotype inference. Table S3.2. Thirty Ancestry Informative Markers (AIMs), genotyped for ancestry estimations. Table S3.3. Fifteen highly variable autosomal single tandem repeats, genotyped for estimations of relatedness. Figure S4.1. Diversity and frequency of the 624 distinct 1.77‐Mb haplotypes deduced by PHASE (874 chromosomes). Figure S4.2. Analysis of STRUCTURE at village level for both Ancestry Informative Markers (above: supervised analysis at k = 3: green: African; red: Amerindian; blue: European) and single tandem repeat markers (below). Figure S4.3. Counts of rs4988235 genotypes per village. Figure S4.4. Boxplot of body mass index per village by sex. [file AHG-83-11-s001.pdf]

Supplementary Materials for 'Adaptation to milking agropastoralism in Chilean goat herders and nutritional benefit of lactase persistence'

MONTALVA, NICOLÁS[1 2 3 †], ADHIKARI, KAUSTUBH[1 4], LIEBERT, ANKE[1], MENDOZA-REVILLA, JAVIER[1 5 ‡], FLORES, SERGIO V.[6], MACE, RUTH [2 §], SWALLOW, DALLAS M.[1]

[1]Research Department of Genetics, Evolution and Environment

University College London

Darwin Building

Gower Street

London WC1E 6BT

United Kingdom

[2]Department of Anthropology

Human Evolutionary Ecology Group

University College London

14 Taviton St

London WC1H 0BW

United Kingdom

[3]Departamento de Antropología

Facultad de Ciencias Sociales y Jurídicas

Universidad de Tarapacá

384 Calle Cardenal Caro

Arica

Chile

[†] **Present address:** Society and Health Research Centre, Universidad Mayor, Santiago, Chile.

[‡] **Present address:** Human Evolutionary Genetics, Institut Pasteur, 75015 Paris, France

[§] **Present address:** Key Laboratory of Arid and Grassland Ecology, Lanzhou University, Lanzhou Gansu Province, China.

[4] Department of Cell & Developmental Biology
University College London
Anatomy Building
Gower Street
London WC1E 6BT
United Kingdom

[5] Laboratorios de Investigación y Desarrollo
Facultad de Ciencias y Filosofía
Universidad Peruana Cayetano Heredia
430 Honorario Delgado
Lima 31
Perú

[6] Departamento de Antropología
Facultad de Ciencias Sociales
Universidad de Chile
1045 Av. Capitan Ignacio Carrera Pinto
Nunoa 7800284
Chile

Section 1: Communities of admixed goat herders in semiarid Chile

The agro-pastoralist groups under study are goat herders in a semi-arid region of Chile, who were selected as a model population because of their pastoralist livelihood, their high dependency on livestock and milk, and their mixed European/Amerindian ancestry and thus variable lactase persistence status. These groups have developed a set of practices over the last 400 years or so, which have been described by social scientists as a 'notable adaptation to their ecological conditions', such as their system of land management, collective property, and territorial organisation, their reliance on multiple sources of income which includes transhumant pastoralism (Gallardo 2002; Alexander 2008). The groups, locally known as "Agricultural Communities" are 180 scattered communities consisting altogether of around 30,000 people. They collectively own approximately 1,000,000 hectares of land with poor irrigation, little arable land and small carrying capacity. The region where these populations are settled is a transitional zone between the Atacama Desert, one of the driest places on Earth, and the Chilean central valleys. The zone has an average annual rainfall of 100–200 mm and constant threat of droughts. The region of Coquimbo, where most of these communities are settled, is a narrow area between the Pacific Ocean and the Andes Mountain Range, covering around 40,000 km^2 extending from 29°S to 32°S around meridian 71°W. Terrain is defined by a pronounced slope of incremental altitude towards the Andes (see Figure S1). This area has west-east oriented mountain ridges below 3,000 metres, and is cut by the three main rivers, named, from north to south: Elqui, Limarí and Choapa which lend their names to the surrounding valleys.

History

From 1470 until the arrival of Europeans in 1537, local native groups of Llama herders in the Coquimbo Region were invaded, and eventually conquered, by the Inca Empire. The native population was likely to have been diminished by this invasion, but there were still native settlements by the time of the first contacts with Spanish groups. Shortly after Spanish arrival the pandemic spread of diseases among Native Americans led to further decline. As in most of Latin America, European migrants were almost all male, so a fast process of admixing started.

The main activity in the southern part of the Spanish Empire was mining and included he 'Norte Chico' for which the region of Coquimbo became the main source of food and fuel for the people working in the mines. It was during this period that livestock and cattle were introduced (Gallardo, 2002). All this led to further land deterioration due to intensive farming, overgrazing, and logging. There are several competing historical hypotheses to explain the origin of the system of communal

property in the area (summarised by Gallardo 2002), as opposed to the development of the large Haciendas and Ranchos developed elsewhere in Spanish America, but it is generally agreed that by the end of the 18th century land deterioration made the system of large-scale production counterproductive. Since then, agriculture gradually lost its economic importance in favour of livestock rearing; and the colonial system of land tenure, economy and subsistence changed to the organisation distinctive of the Agricultural Communities today.

Population size

The population sizes for each collection site as obtained from a special bulletin of the Chilean National Institute of Statistics (INE) devoted to the Agricultural Communities of the Coquimbo region (Vergara et al., 2005) is shown in Table S1. These data are based on the Chilean National Census of 2002, which although outdated, is the most recent reliable data source available[**].

Table S1. Population of sampled sites, total population, and number of participants in the study (n).

Community	Census population	n
Barraza	344	41
Canela Baja	1,426	164
Canelilla Ovalle	114	23
Castillo Mal Paso	295	63
El Espinal	43	15
Gualliguaica	114	7
Huentelauquen	352	42
La Calera	186	49
La Polvada	139	7
Monte Patria	888	40
**Total (sampled sites)**	**3,901**	**451**
**Total (all Agricultural Communities)**	**38,604**	**451**

[**] A national census was conducted in 2012, but has been seriously questioned on methodological grounds. (BBC News 2013. http://www.bbc.co.uk/news/world-latin-america-23611210.

Economy and subsistence

In the last two hundred years the main means of subsistence has been livestock rearing. Between 80–90% of the total lands of a community is unenclosed common fields, used collectively for livestock grazing. Goats are the main animal reared, followed by sheep, with cattle found only in some of the southernmost communities. The herders sell goat meat, skin, and cheese, with cheese being a particularly important source of income. Most herders plan their animals' births once a year, generally around September.

By custom, and until recently, during dry years, the whole family crossed the Andes to the Argentinian side of the mountains with their livestock, and stayed there from November to March, (the summer months), when melted snow makes new pastures available for grazing. Cheese was made at their summer dwellings, to be sold on their way back to the winter settlements and goats were milked during the journey. During this trip, meals were comprised mainly of goat's meat, cheese, and milk taken with 'churrasca' (flat unleavened bread made with flour, butter and water, roasted in the coals of a campfire). In addition to pure fresh milk, milk was also consumed with 'mate' (a common herbal infusion in South America), and as 'cocho' (toasted wheat flour mixed with milk and eaten as porridge).

This transhumant pastoralism has now become somewhat more restricted, the distance travelled being less. This is partly because selling and production of cheese is becoming more difficult because of hygiene regulations (Alexander, 2008) and partly because school is compulsory. Today most of the milk consumed is industrially produced cow's milk bought in stores, and milk consumption is similar to that of other Chilean rural populations (see Fernández et al. 2015). Nonetheless, at least until very recently, milk and dairy products were the dominant part of the daily intake of food in these groups for around five months every year, a diet that is somewhat surprising in view of the historic lactase non-persistence in Amerindians.

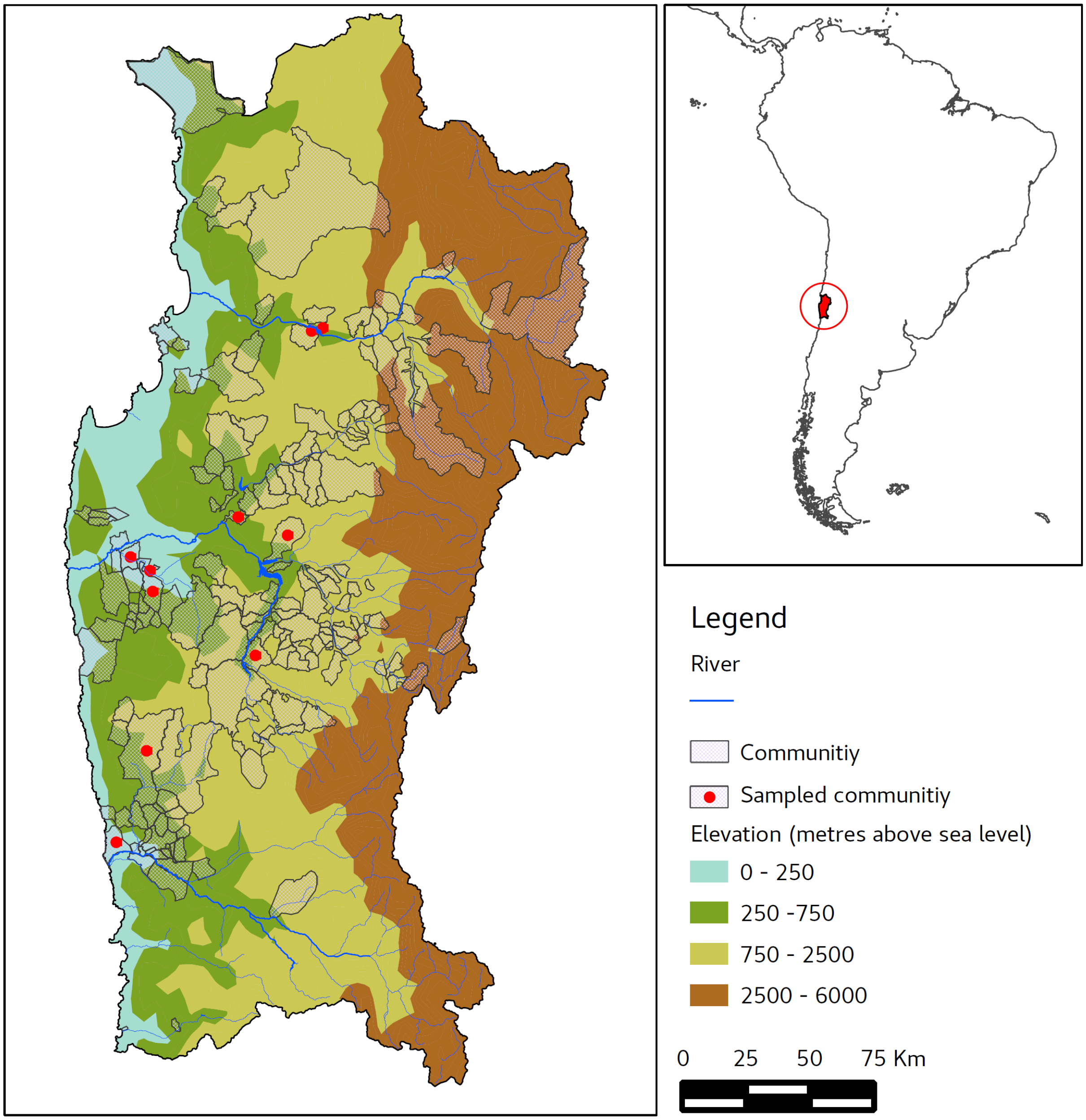

Figure S1. Left**: Map of the Coquimbo Region showing the location of the Agricultural Communities,** showing elevation, and the three main rivers with their associated valleys. Right: **Location of the Coquimbo Region in South America.** The communities from which the collections were made are indicated with red dots.

Section 2: Recruitment and Demographic Profile

The study volunteers were recruited from the Agricultural Communities of the Chilean region of Coquimbo in South America, a set of populations featuring milking pastoralism of recent adoption.

Data collection

451 adult volunteers were recruited in 9 villages and hamlets from the Coquimbo region (Table S1). They were invited to participate after being fully informed about the project and the security of their personal data, and written consent was obtained for each of the participants who agreed to have their data analysed as part of this study. A demographic profile based on our sample of 451 participants can be found in Table S2.

As is normally the case in anthropological studies in isolated areas, villages were selected strategically according to population size and accessibility, and participants were all volunteers in compliance with ethical standards. Therefore, biases from sampling relatives and self–reported lactose intolerant individuals could not be avoided at this stage, but genetic methods, described in the main text, were adopted to account for their effects.

Local people from each community were approached during the first days of fieldwork before any recruitment, in order to let them know about our work there. Throughout this phase informal interviews were conducted to collect and record general information about the area, trends in migration and demography, dietary habits, goat rearing practices, milking, and milk processing.

Afterwards suitable prospective participants were approached, the details of our project were explained, and information sheets were provided. Suitable meeting space in communal venues was arranged in each village to interview those who agreed to participate.

For strategic reasons (time and complexity of the tests) all the LTT phenotype data collection (n=41) was done in the same village (Barraza) selecting non-smoker volunteers who were not currently undergoing treatment with antibiotics and prepared to undergo an overnight fast.

Questionnaires administered by the first author and trained interviewers were used to collect information as to the length of residency, residency at birth, place of origin of parents and grandparents, details of children, their birth-places, and date of death when applicable. Ownership

of assets, livestock, communal rights, access to services and milk product consumption were also recorded. Participants reported whether they or their household have access to certain goods and services (such as tap water, electricity, etc. See Table S2). These data were used to measure wealth by processing all assets using a multiple correspondence analysis, to get a factorial weight based on the contribution to the inertia at the first principal axis of each item (Asselin and Anh 2008). Interviews were carried out at local communal facilities (e.g. sports clubs, community associations, etc.), or at the house of the interviewed volunteer, according to their preference.

Quantities and frequencies of glasses of milk consumed were used to estimate milk consumption as numbers of glasses per day. The paper records were entered into a database using EpiData Entry (Lauritsen and Bruus 2008) which provided facilities for tailored consistency checks, automatic backups, random double–entry verification, and encryption. The demographic profile of the sample is shown in Table S2.1and S2.2, and Figures S2.1 to S2.8.

Lactose Tolerance Testing

Breath hydrogen levels were measured using a breath hydrogen monitor (MicroH_2 Micromedical Ltd.). After an overnight fast and measuring baseline levels, subjects were given a load of 50 g of lactose in 250 ml of water. Afterwards, breath hydrogen levels were recorded at intervals of 30 minutes. A given test was stopped after two sustained increments of 20 ppm above baseline, when lactase persistence status was assessed as a lactose non–digester. Individuals showing no substantial rise in breath hydrogen after 3 hours were classified as lactose digesters. The phenotypes of subjects showing fluctuating levels of breath hydrogen were classified as indeterminate and those whole failed to produce breath hydrogen throughout the test as hydrogen non-producers (i.e. do not have appropriate hydrogen-producing colonic bacteria).

Table S2.1. Demographic profile of 451 sample donors from nine villages. Missing data were excluded, and percentages were rounded to the nearest integer.

Category	n	%
**Sex**		
Male	153	34
Female	298	66
**Number of grandparents born outside the communities**		
None	327	73
1	43	10
2	39	9
3	12	3
All	29	6
**Number of children (parity – both sexes)**		
0	66	15
1	51	11
2	111	25
3	80	18
4	53	12
5	30	7
6	23	5
7+	34	8
**Access to goods/services**		
Tap water	413	92
Electricity	436	97
Ceiling	439	98
Floor	429	96
Water heater	283	63
Washing machine	386	86
Fridge	413	92
Television	429	96
Computer	173	39
Motor vehicle	169	38

Table S2.2. Sample information by sex. Missing data were excluded. Percentages are rounded to one decimal place

	Males		Females		Both	
	n	%	n	%	n	%
**Age**						
18-29	16	10.6	32	10.9	48	10.8
30-39	17	11.3	53	18	70	15.7
40-49	24	15.9	58	19.7	82	18.4
50-59	32	21.2	53	18	85	19.1
60-69	20	13.2	45	15.3	65	14.6
70+	42	27.8	53	18	95	21.3
**European Ancestry**						
< 0.2	1	0.7	2	0.7	3	0.6
0.2-0.39	33	22.8	77	26.4	110	25.2
0.4-0.59	86	59.3	160	54.8	246	56.3
0.6-0.79	24	16.6	53	18.2	77	17.6
> 0.8	1	0.7	0	0	1	0.2
**Milk consumption (cups 250cc)**						
None	28	18.4	57	19.2	85	18.9
Less than 2	115	75.7	221	74.4	336	74.8
2-3	5	3.3	18	6.1	23	5.1
More than 3	4	2.6	1	0.3	5	1.1
**Height (cm)**						
< 150	3	0.2	66	22.2	69	15.4
150-159	16	10.6	178	59.9	194	43.3
160-169	71	47.7	51	17.2	122	27.2
170-179	55	36.4	1	0.3	56	12.5
> 180	6	4	1	0.3	7	1.6
**Weight (kg)**						
<40	0	0	1	0.3	1	0.2
40-59	10	6.6	64	21.6	74	16.6
60-79	82	54.3	180	60.8	262	58.6
80-99	56	37.1	47	15.9	103	23
>99	3	2	4	1.4	7	1.6
BMI						
< 18.5	1	0.7	2	0.7	3	0.7
18.5-24.9	36	23.8	57	19.3	93	20.8
25-29.9	76	50.3	118	39.9	194	43.4
30-34.9	34	22.5	86	29.1	120	26.8
> 35	4	2.6	33	11.1	37	8.3
**Number of Children ever Born**						
None	30	19.7	36	12.2	66	14.7
1-2	53	34.9	109	36.8	162	36.2
3-4	37	24.3	96	32.4	133	29.7
5-6	20	13.2	33	11.1	53	11.8
> 7	12	7.9	22	7.4	34	7.6
**Number of deceased children**						
None	133	87.5	249	83.8	382	85.1
1	14	9.2	40	13.5	54	12
2	2	1.3	6	2	8	1.8
3	3	2	1	0.3	4	0.9
4	0	0	1	0.3	1	0.2

.

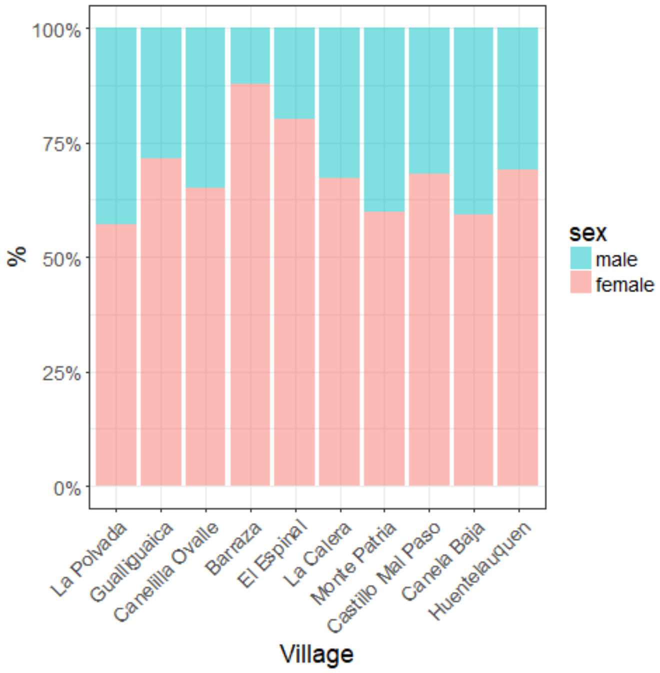

Figure S2.1. Percentage of participants by sex in each village.

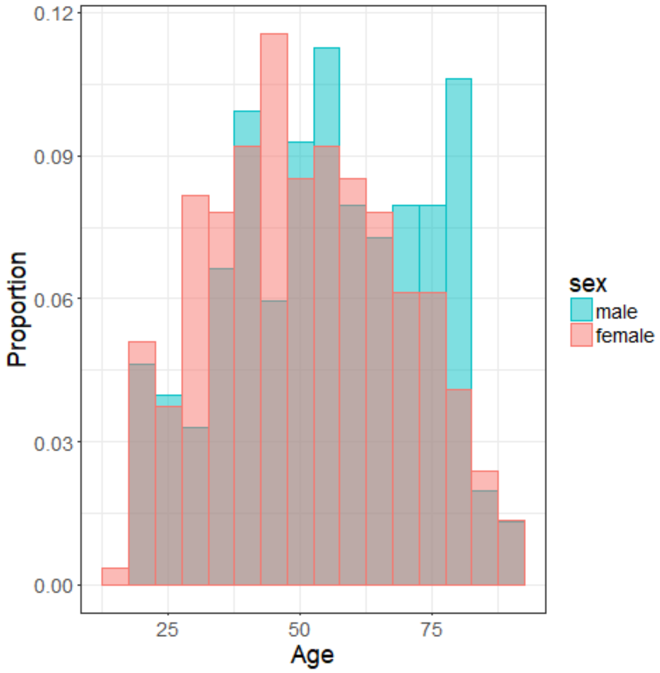

Figure S2.2. Age of participants by sex. *All samples:* Mean = 52.4, s.d. = 18, range = 18 – 92. *Females:* Mean = 51, s.d. = 17.7, range = 18 – 92. *Males:* Mean = 55.2, s.d. = 18.3, range = 18 – 92.

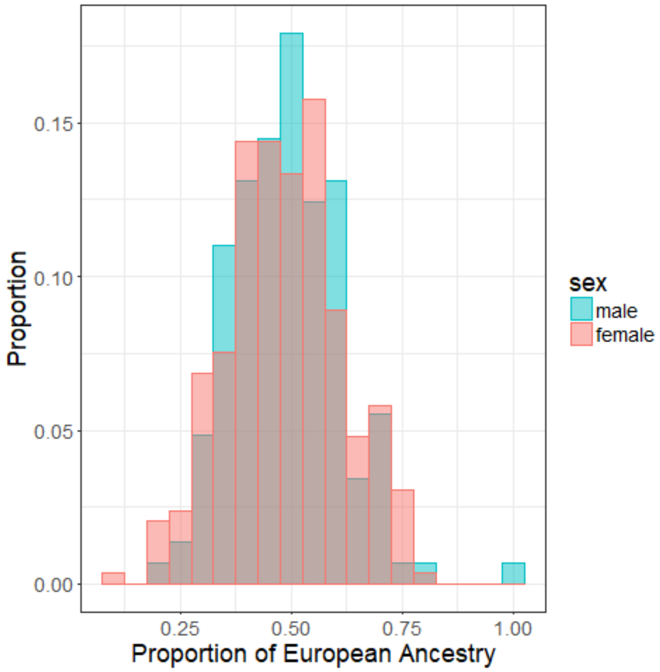

Figure S2.3. Proportion of European ancestry of participants by sex. *All samples:* Mean = 0.48, s.d. = 0.126, range = 0.09 – 1. *Females:* Mean = 0.48, s.d. = 0.127, range = 0.09 – 0.78. *Males:* Mean = 0.49, s.d. = 0.123, range = 0.198 – 1

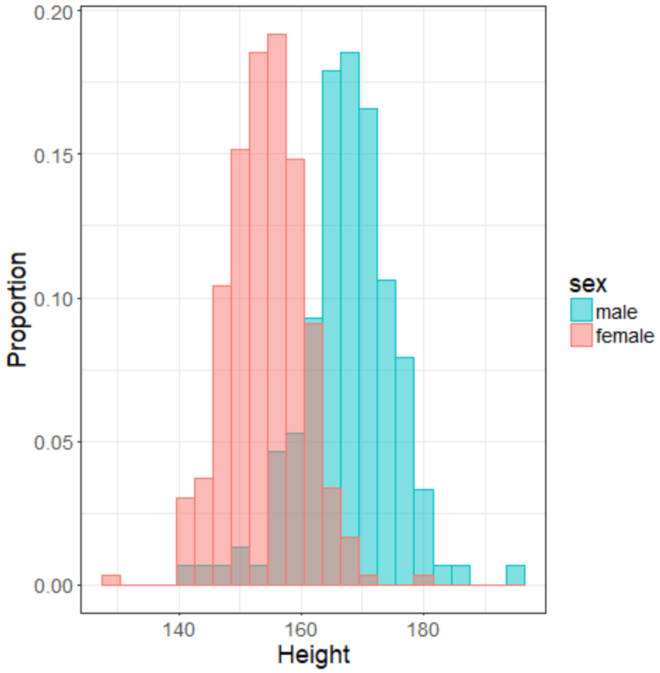

Figure S2.4. Height of participants by sex. *All samples:* Mean = 158.8, s.d. = 9.26, range = 130.2 – 195. *Females:* Mean = 154.5, s.d. = 6.2, range = 130.2 – 180. *Males:* Mean = 167.8, s.d. = 7.65, range = 139.8 – 195.

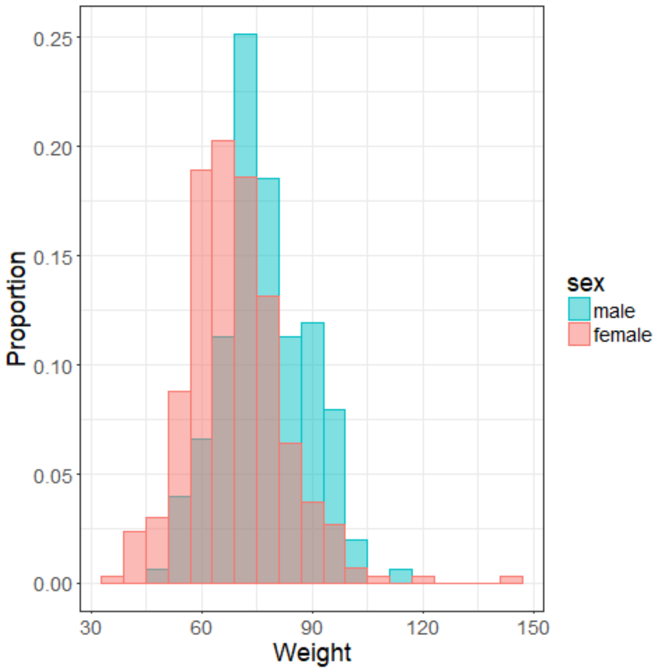

Figure S2.5. Weight of participants by sex. *All samples:* Mean = 69.2, s.d. = 13.3, range = 37.4 – 146.4. *Females:* Mean = 69.22, s.d. = 13.11, range = 37.4 – 146.4. *Males:* Mean = 77.27, s.d. = 12.07, range = 48.7 – 113.6.

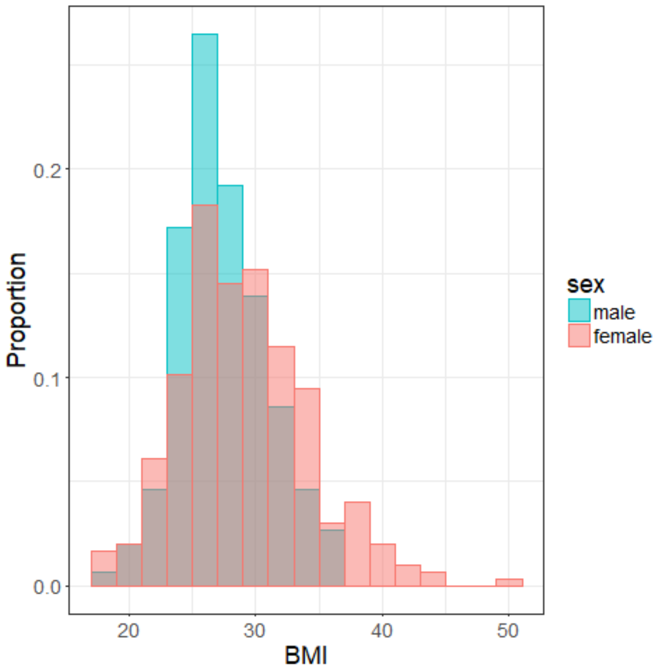

Figure S2.6. BMI of participants by sex. *All samples:* Mean = 28.5, s.d. = 4.69, range = 17.84 – 50.5. *Females:* Mean = 29.1, s.d. = 5.1, range = 18.2 – 50.5. *Males:* Mean = 27.4, s.d. = 3.5, range = 17.84 – 36.

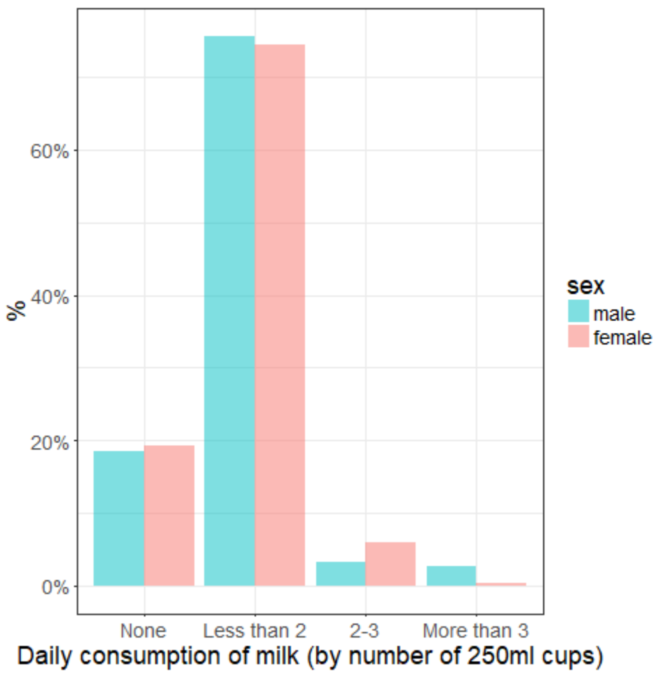

Figure S2.7. Milk consumption of participants by sex. *All samples:* Mean = 0.56, s.d. = 0.73, range = 0 – 4. *Females:* Mean = 0.55, s.d. = 0.68, range = 0 – 3.25. *Males:* Mean = 0.6, s.d. = 0.82, range = 0 – 4.

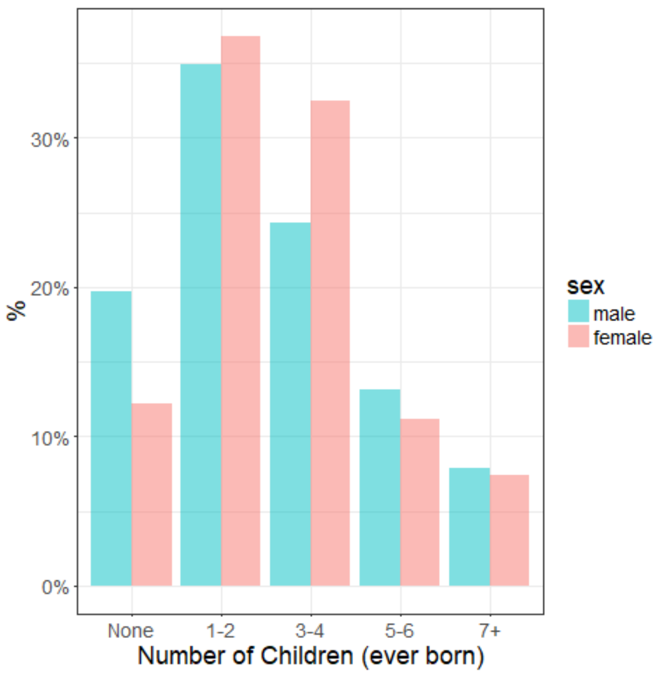

Figure S2.8. Children ever born of participants by sex. *All samples:* Mean = 2.93, s.d. = 2.4, range = 0 – 16. *Females:* Mean = 2.98, s.d. = 2.26, range = 0 – 15. *Males:* Mean = 2.81, s.d. = 2.64, range = 0 – 16.

Section 3: Genetic markers used in this study

Samples of buccal cells were collected from cotton swabs to perform DNA extractions by modified versions of methods based on phenol/chloroform (Freeman et al. 2003) and salting out precipitation (Quinque et al. 2006). A segment of 706 bp of the *LCT* enhancer region (*MCM6*, intron 13) was amplified by PCR using primers MCM6i13 and MCM6778 described by Ingram et al. (2007). Samples were sequenced in both directions on an ABI 3730xl DNA Analyzer (Applied Biosystems) by the UCL Centre for Comparative Genomics, using the Sanger Method.

In addition, each individual was also typed for a set of 15 autosomal STRs, 30 SNPs used as Ancestry Informative Markers (AIMs), and 27 SNPs in chromosome 2 surrounding −13,910C>T, to be used for haplotype inference and estimations of whole-genome and local ancestry. The 15 autosomal STR *loci* were obtained using a kit designed for forensic identification (Promega PowerPlex 16 HS).

The panel of 30 AIMs used was described and validated by Ruiz-Linares et al. (2014). This panel was especially constructed to estimate the three main continental ancestry components in Latin Americans, using markers whose allele frequencies are highly differentiated between the three major ancestry components in Latin America (i.e. Amerindian, European and African). Ancestry estimates using these 30 AIMs have ~70% correlation with ancestry estimated from a genome-wide SNP chip data with ~50,000 SNPs (after LD pruning) (Ruiz-Linares et al. 2014). The AIMs were selected as a compromise between the cost of genotyping more markers and gain in precision. In the Ruiz-Linares et al. 2014 paper, another proposed set of AIMs for Latin Americans (Galanter et al. 2012) was also compared. Using 152 SNPs (i.e. 5 times the number of proposed AIMs), the gain of accuracy was only 15% measured by correlation with ancestry estimates from chip data.

The panel of SNPs on chromosome 2 was selected by choosing the most informative 27 of 36 SNPs used for haplotype analysis for our parallel worldwide population study (Liebert et al. 2017) and cover 1.77Mb. The 36 SNPs had been selected such that the physical distance between them was on average 50kb, but were placed further apart in regions of 100% LD as assessed using linkage disequilibrium unit maps based on HapMap populations. The 27 SNPs used in this study (Table S3.1) included 2 SNPs (rs3754689 and rs2278544) useful for identification of the core haplotypes described by Hollox et al. (2001) and rs182549 located at −22kb known to be in very high LD with

−13,910C>T. Both sets of SNPs (30 AIMs and the 27 SNPs on chromosome 2) were typed by LGC Genomics (Hoddesdon, UK) using KASP chemistry.

Table S3.1. Twenty-seven SNPs surrounding *LCT* enhancer region on Chromosome 2, genotyped for haplotype inference. Minor allele frequencies and other data obtained from Ensembl Genome Browser (Flicek et al., 2014) and 1,000 Genomes Project (The 1000 Genomes Project Consortium, 2012). SNPs included in the analysis of delta ancestry marked in bold with an asterisk.

rs number	Position (build 37)	Ref/Alt	Ancestral	Minor allele (worldwide)	MAF (worldwide)
rs1446525	135637847	G/A	A	G	0.288
**rs4954209***	135737908	G/T	T	T	0.348
**rs2874739***	135818907	C/T	T	T	0.347
rs1869829	135877562	A/G	G	T	0.385
rs2305248	135928312	A/G	G	G	0.343
**rs1900741***	136002500	C/T	T	C	0.431
rs1561277	136092061	C/A	A	C	0.264
rs6709132	136232572	A/G	G	G	0.211
**rs3806502***	136288273	C/T	T	A	0.327
**rs4954265***	136324225	A/G	G	G	0.27
**rs961360***	136393658	A/G	A	C	0.315
**rs4954278***	136408291	C/T	C	T	0.181
rs2278544	136546110	A/G	A	G	0.492
rs2304370	136561735	G/A	G	T	0.254
**rs3754689***	136590746	C/T	C	T	0.339
**rs182549***	136616754	C/T	C	T	0.234
**rs309152***	136657252	T/C	C	G	0.321
**rs309137***	136765951	T/C	C	T	0.376
rs2090660	136818719	C/T	C	A	0.269
**rs12691874***	136880474	G/A	G	A	0.339
**rs953387***	136907170	A/C	A	T	0.46
**rs12465599***	137074850	A/G	G	G	0.439
rs6715450	137121731	G/A	A	A	0.346
rs543721	137161557	G/T	G	T	0.411
rs12618749	137205474	C/T	C	T	0.228
**rs580879***	137314139	C/T	T	A	0.257
rs6711718	137407012	T/C	T	C	0.451

Table S3.2. Thirty Ancestry Informative Markers (AIM), genotyped for ancestry estimations. Minor allele frequencies (MAF) showed in the last three columns refer to frequencies in African, Amerindian and European populations, and were provided by the laboratory of Professor Andrés Ruiz Linares at UCL GEE (Ruiz-Linares et al. 2014). Other data obtained from Ensembl Genome Browser (Flicek et al. 2014) and 1,000 Genomes Project (The 1000 Genomes Project Consortium, 2012).

rs number	Location band	Ref/Alt	Ancestral	Minor Allele (worldwide)	Continental MAF		
					Africa	Americas	Europe
rs1544450	1p13.1	G/T	T	T	0.947	0	0.089
rs1834619	2p24.2	G/A	G	A	0	0.975	0.037
rs356652	2q11.2	T/G	T	G	0	0.933	0.067
rs260690	2q12.3	C/A	C	C	0.642	0.963	0.045
rs2176046	2q37.3	G/A	G	A	0.015	0.934	0.06
rs10510511	3p24.3	G/T	G	T	0	0.916	0.023
rs3870336	3p21.31	G/A	G	A	0.089	0.936	0.085
rs10935320	3q23	T/C	T	C	0.154	0.979	0.104
rs11725412	4p14	A/G	A	A	0.21	1	0.06
rs10037656	5p15.32	A/G	A	G	0.337	0.98	0.099
rs4145160	5q33.2	G/A	G	A	0.095	0.912	0.067
rs1559163	5q33.2	A/G	A	G	0	0.853	0.023
rs2042314	5q35.1	C/T	C	T	0.139	0.999	0.146
rs12662498	6p12.1	G/A	G	A	0.012	0.98	0.07
rs17086231	6q25.3	C/T	C	T	0.018	0.943	0.117
rs6464749	7q35	A/G	A	G	0.893	0	0.05
rs7018273	8q21.13	A/G	G	G	0.858	0	0.017
rs12347078	9p24.3	A/C	A	C	0.876	0	0.035
rs734241	10q25.3	G/A	G	A	0.044	0.989	0.065
rs174570	11q12.2	C/T	C	T	0.006	0.997	0.111
rs7134749	12q13.12	T/C	T	C	0.21	0.898	0.027
rs2052386	12q15	G/A	G	A	0.077	0.929	0.095
rs1849384	12q21.31	A/C	C	C	0.973	0	0.082
rs4769128	13q12.11	C/T	C	T	0.154	0.988	0.13
rs1243370	14q11.2	T/C	T	C	0.24	0.919	0.055
rs2719921	15q11.2	G/A	A	A	0.876	0	0.033
rs1197062	17q23.2	T/G	G	G	0.891	0	0.057
rs717225	19q13.2	A/G	G	G	0.885	0	0.008
rs6119879	20q11.21	C/T	C	T	0.686	0	0.84
rs2426552	20q13.2	C/T	T	T	0.834	0	0.008

Table S3.3. Fifteen highly variable autosomal STR, genotyped for estimations of relatedness.

	Location band	Alleles (by number of repeats)
TPOX	2p25.3	6-13
D3S1358	3p21.31	12-20
FGA	4q28	16-46
D5S818	5q23.2	7-16
CSF1PO	5q33.1	6-15
D7S820	7q21.11	6-14
D8S1179	8q24.13	7-18
TH01	11p15.5	4-13
Vwa	12p13.31	10-22
D13S317	13q31.1	7-15
PentaE	15q26.2	5-24
D16S539	16q24.1	5-15
D18S51	18q21.33	8-27
D21S11	21q21.1	24-38
PentaD	21q22.3	2-17

Section 4: Haplotypic background and Assessment of structure between villages.

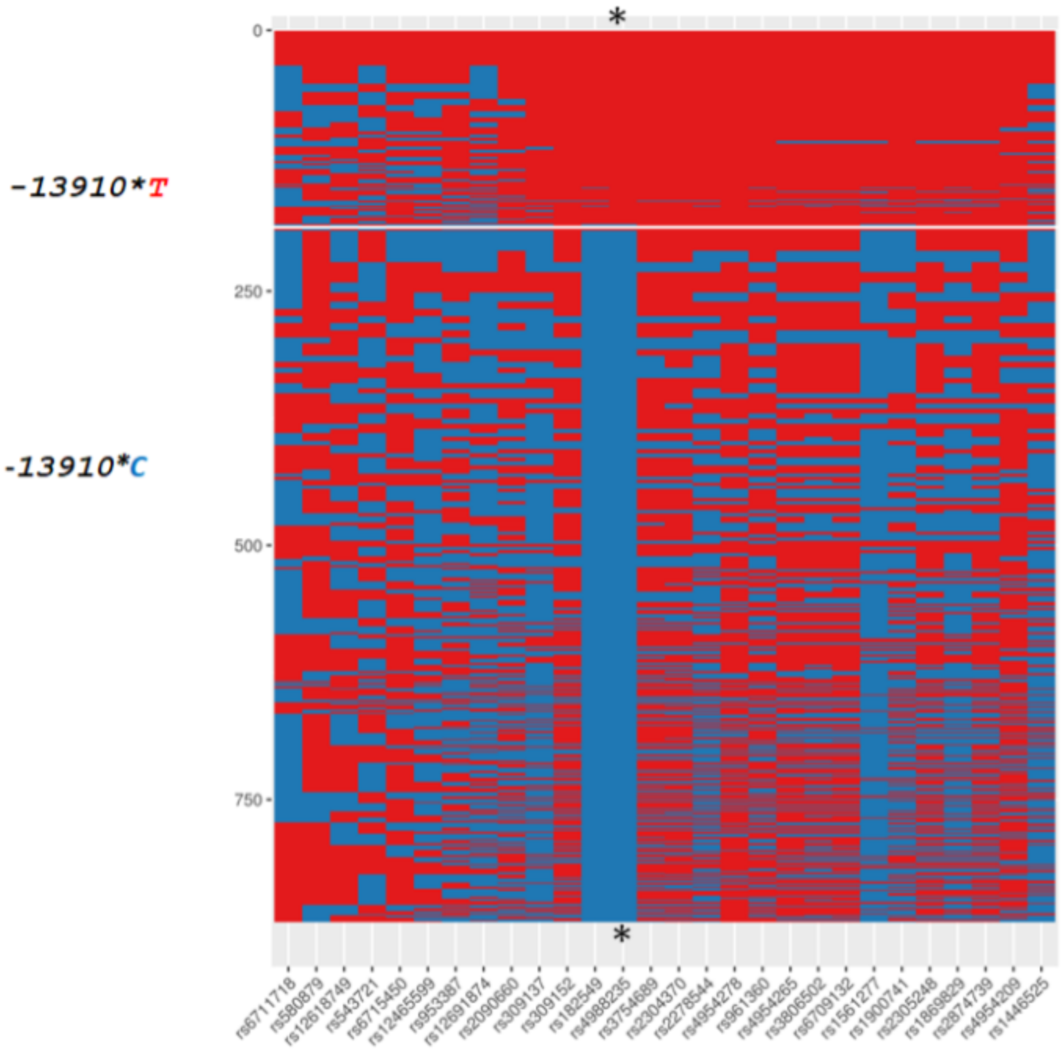

Figure S4.1. **Diversity and frequency of the 624 distinct 1.77 Mb haplotypes deduced by PHASE (874 chromosomes)**. Ordered in haplotype frequency for the ancestral (red) and derived alleles (blue) of rs4988235 (−13,910C>T, marked with an asterisk). The most frequent haplotype occurs 10 times. The 34 most frequent haplotypes are the same as the most frequent '**A**' core haplotype detected in Europeans with the same markers.

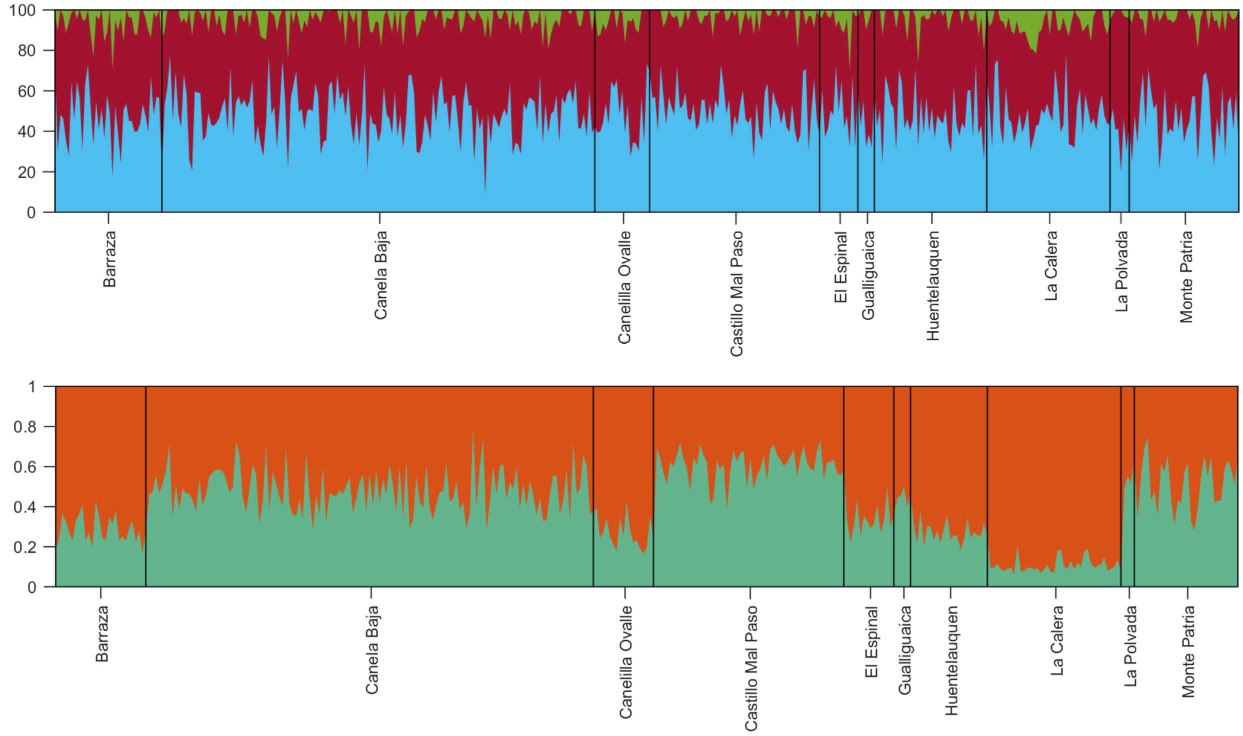

Figure S4.2. **Analysis of STRUCTURE at village level for both AIMs (above: supervised analysis at *k* = 3: green. African, red: Amerindian, blue: European) and STR markers (below).** Note that the STR markers do not reflect the same clustering as the AIMs and are clearly not detecting continental ancestry, but more likely more recent geographic differences in ancestry, which are likely to be the product of increased relatedness within villages.

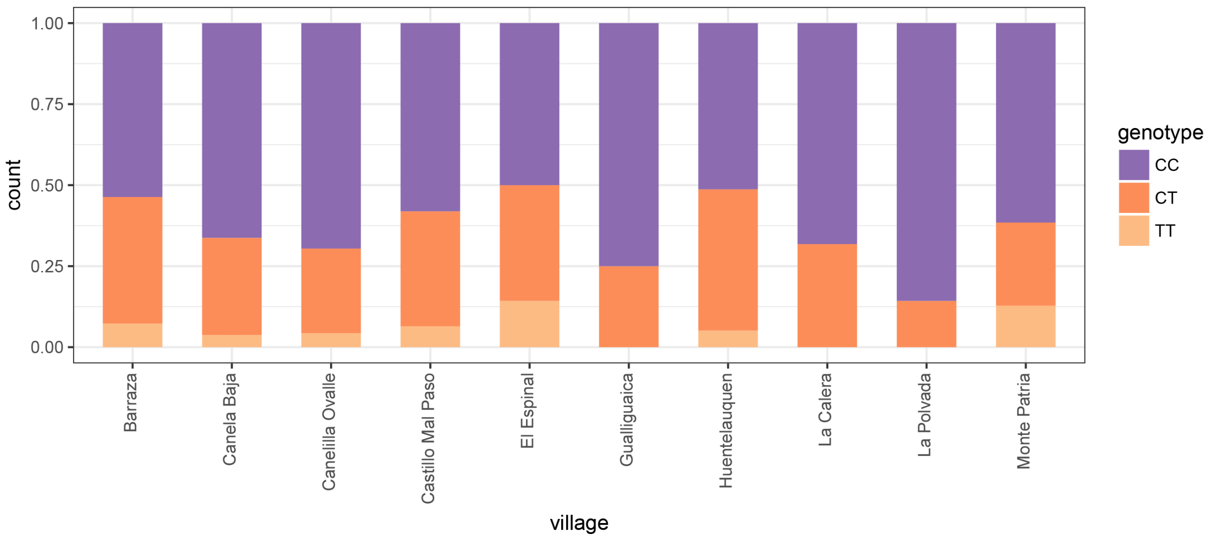

Figure S4.3. Counts of rs4988235 genotypes per village. There is no evidence of significant stratification of *−13,910*T* carriers between villages, and therefore village-level covariates were not used in regression models. Note that the villages Gualliguaica and La Polvada have very small sample sizes (See Table S1.)

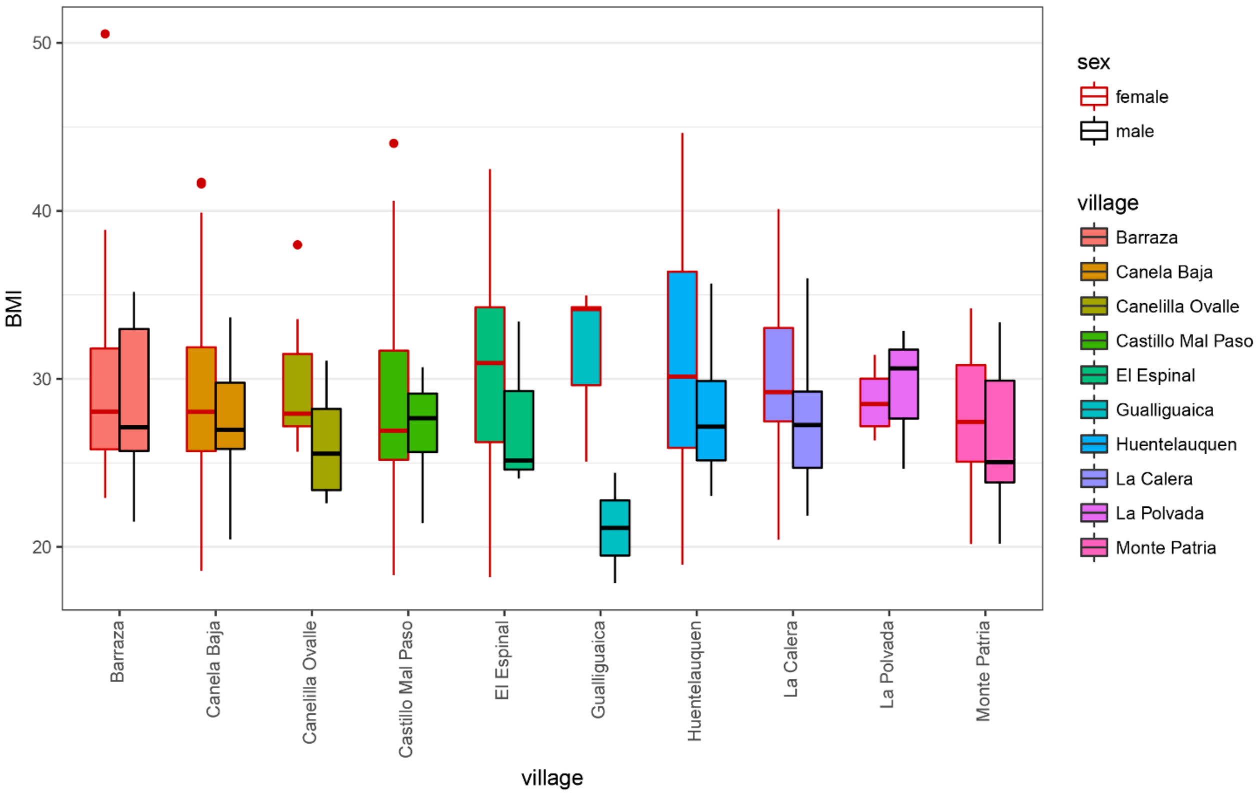

Figure S4.4. Boxplot of Body Mass Index per village by sex. Note that females (red bars on left) have higher BMI than males in all but two villages. Note that the villages Gualliguaica and La Polvada have very small sample sizes (See Table S1.)

Section 5: Local Ancestry

Haplotype-based local ancestry analysis

In an attempt to further validate this analysis using whole-genome high-density genotype data from the 1000 Genomes database (The 1000 Genomes Project Consortium 2012), the admixture-based analysis above was replicated with haplotype-based local ancestry estimates using high-density genotype data. As the high-density SNP panel is able to resolve all the three continental ancestries (Maples et al. 2013; Rishishwar et al. 2015), this analysis could be performed on all three mainland Latin American populations: in addition to Mexicans from Los Angeles (MXL) and Peruvians from Lima (PEL), Colombians from Medellin (CLM) who have substantial African ancestry could also be used. All SNPs with >1% minor allele frequency (MAF) in the Latin American samples were retained. Samples were merged with the reference panel of European, African and Amerindian samples (Ruiz-Linares et al. 2014), retaining a total of 546,780 SNPs, of which 403 were in the Lactase region being studied.

For whole-genome ancestry estimation, the merged dataset was LD-pruned using PLINKv1.9 (resulting in 150,858 SNPs being retained), and supervised Admixture (k = 3) was performed. For local ancestry estimation, haplotype-based method RFMix (Maples et al. 2013) was used to provide higher accuracy in ancestry assignments using LD, and also meaning that more SNPs can be used towards the inference, as LD-based pruning is not required.

The genotype data (of both admixed and reference individuals together) was first phased using the software SHAPEIT2 (Delaneau et al. 2012) with default parameters. Local ancestry assignments were performed using the software RFMix to infer local continental ancestry in the subset of phased admixed individuals.

As reference continental panels, we used 175 Amerindians (from Chacon-Duque et al. 2018), 107 IBS (Iberian populations in Spain) and 101 YRI (Yoruba in Ibadan, Nigeria) individuals from The 1000 Genomes Project. We ran RFMix with the phase correction feature enabled and performed two rounds of the EM algorithm. We used the default settings except the number of reference haplotype per tree node, which was set to 5. This was done to take into account unbalanced reference panel sizes in the random forest algorithm, as recommended by Maples et al. 2013. RFMix assigns local continental ancestry to each allele of each admixed haplotype, allowing for errors in genotyping,

slight admixture in the reference samples, etc. For each locus across both haplotypes the posterior probabilities were converted to the most likely ancestry and aggregated to estimate the proportion European ancestry for that person.

Average local European ancestry (obtained via RFMix) of the Lactase region under study (1Mb either side of rs4988235) was compared to the average genome-wide European ancestry (obtained via Admixture) using the same one-sided Wilcoxon signed rank test. All p-values were again non-significant: 0.4583 for MXL, 0.3032 for PEL, and 0.4467 for CLM.

Power calculations

In order to determine whether the Admixture Method to determine local ancestry had sufficient Power to detect a 3% difference in local ancestry (delta ancestry) in the control populations, simulations were done in which the sample numbers and ancestry proportions and distributions mirrored those groups. Power was estimated to be 0.2683 for MXL and 0.8034 for PEL.

Using the haplotype based method RFMix to determine local ancestry, simulations show that an increase of 3% European ancestry locally would be detected with power 0.3836 for MXL, 0.4750 for PEL and 0.6562 for CLM respectively. The variations in power depend on the varying sample sizes of the three groups.

Section 6: Summary of samples by method.

Collected data:

- **DNA samples:** 451 samples, 437 successfully sequenced.
- **Height and weight:** 447.
- **Questionnaires:** 451.
- **Lactose Tolerance Tests:** 41.

Analyses:

Phenotype Genotype association:

- Sample size: 41
- Data used: 41 Genotypes from Sanger sequences and 41 Lactose Tolerance Tests.

Haplotypic background:

- Sample size: 437
- Data used: 437 DNA samples genotyped for 27 SNPs in Table S3.1

Relatedness:

- Sample size: 351
- Data used: 351 DNA samples genotyped for all 15 STR loci in Table S3.3.

Ancestry:

- Sample size: 408
- Data used: 408 DNA samples with less than 20% failure rate when genotyping for 30 AIMs in Table S.3.2.

Association with milk consumption:

- Sample size: 437
- Data used: 437 DNA samples successfully genotyped for *−13,910*T* at rs4988235 and with paired data of milk consumption obtained from questionnaires.

Association with BMI

- Sample size: 329
- Data used: 329 individuals with complete data for BMI (obtained from height and weight measures in the field) and variables included in the model: genotype for *−13,910*T* at rs4988235 (from sequences), ancestry proportions (from 30 AIMs), and age (from questionnaires).

Association with number of children

- Sample size: 415
- Data used: 415 individuals with complete data for all the variables included in the model: number of children, age, milk consumption, and sex (from questionnaires), wealth (calculated from access to goods and services as described in Methods), BMI (from height and weight), and ancestry proportions (from 30 AIMs).

Local Ancestry Analysis

- Sample size: 408
- Sample size for control samples: 55 MXL (Mexicans from Los Angeles, USA), 76 PEL (Peruvians in Lima, Peru)
- Data used: 30 AIMs for whole-genome continental ancestry; 16 SNPs in the *LCT* region for local continental ancestry.

Local Ancestry Analysis with high-density genotypes

- Sample size for control samples: 55 MXL (Mexicans from Los Angeles, USA), 76 PEL (Peruvians in Lima, Peru), 93 CLM (Colombians from Medellin, Colombia)
- Data used: 150,858 SNPs for whole-genome continental ancestry; 403 SNPs in the *LCT* region for local continental ancestry.

Supplementary Material References

Alexander WL (2008) Resiliency in hostile environments: a comunidad agrícola in Chile's Norte Chico. Lehigh University Press, Cranbury

Asselin L-M, Anh VT (2008) Multidimensional Poverty and Multiple Correspondence Analysis. In: Quantitative Approaches to Multidimensional Poverty Measurement. Palgrave Macmillan UK, London, pp 80–103

Chacon-Duque JC, Adhikari K, Fuentes-Guajardo M, et al (2018) Latin Americans show wide-spread Converso ancestry and the imprint of local Native ancestry on physical appearance. bioRxiv 252155 . doi: 10.1101/252155

Delaneau O, Zagury J-F, Marchini J (2012) Improved whole-chromosome phasing for disease and population genetic studies. Nat Methods 10:5–6 . doi: 10.1038/nmeth.2307

Fernández CI, Montalva N, Arias M, et al (2015) Lactase non-persistence and general patterns of dairy intake in indigenous and mestizo chilean populations. Am J Hum Biol. doi: 10.1002/ajhb.22775

Flicek P, Amode MR, Barrell D, et al (2014) Ensembl 2014. Nucleic Acids Res 42:D749-55 . doi: 10.1093/nar/gkt1196

Freeman B, Smith N, Curtis C, et al (2003) DNA from buccal swabs recruited by mail: evaluation of storage effects on long-term stability and suitability for multiplex polymerase chain reaction genotyping. Behav Genet 33:67–72

Galanter JM, Fernandez-Lopez JC, Gignoux CR, et al (2012) Development of a panel of genome-wide ancestry informative markers to study admixture throughout the Americas. PLoS Genet 8:e1002554 . doi: 10.1371/journal.pgen.1002554

Gallardo G (2002) Communal Land Ownership in Chile: The Agricultural Communities in the Commune of Canela, Norte Chico (1600-1998) (International Land Management Series). Ashgate, Aldershot

Hollox EJ, Poulter M, Zvarik M, et al (2001) Lactase haplotype diversity in the Old World. Am J Hum Genet 68:160–172 . doi: 10.1086/316924

Ingram CJE, Elamin MF, Mulcare C, et al (2007) A novel polymorphism associated with lactose tolerance in Africa: multiple causes for lactase persistence? Hum Genet 120:779–788 . doi: 10.1007/s00439-006-0291-1

Lauritsen J, Bruus M (2008) EpiData 3.1. A comprehensive tool for validated entry and documentation of data. [Software]

Maples BK, Gravel S, Kenny EE, Bustamante CD (2013) RFMix: A discriminative modeling approach for rapid and robust local-ancestry inference. Am J Hum Genet 93:278–288 . doi: 10.1016/j.ajhg.2013.06.020

Quinque D, Kittler R, Kayser M, et al (2006) Evaluation of saliva as a source of human DNA for population and association studies. Anal Biochem 353:272–277 . doi: 10.1016/j.ab.2006.03.021

Rishishwar L, Conley AB, Wigington CH, et al (2015) Ancestry, admixture and fitness in Colombian genomes. Sci Rep 5:12376 . doi: 10.1038/srep12376

Ruiz-Linares A, Adhikari K, Acuña-Alonzo V, et al (2014) Admixture in Latin America: Geographic Structure, Phenotypic Diversity and Self-Perception of Ancestry Based on 7,342 Individuals. PLoS Genet 10:e1004572 . doi: 10.1371/journal.pgen.1004572

The 1000 Genomes Project Consortium (2012) An integrated map of genetic variation from 1,092 human

genomes. Nature 491:56–65 . doi: 10.1038/nature11632
